# FOXO1-driven metabolic reprogramming of hematomal CD8^+^ T cells drives the expansion of perihematomal edema following intracerebral hemorrhage

**DOI:** 10.1038/s41423-025-01363-x

**Published:** 2025-11-14

**Authors:** Jie Lin, Honglei Ren, Youliang Wang, Hanzhi Yu, Zhili Chen, Xintong Yu, Zhuyu Gao, Yan Zheng, Quanhong Wu, Yizhe Zhang, Qijian Lin, Rui Li, Decai Tian, Zhigang Cai, Qiang Liu, Ying Fu

**Affiliations:** 1 Department of Neurology of First Affiliated Hospital, Institute of Neurology, Fujian Medical University, Fuzhou, 350000 China; 2 Department of Neurology, Tianjin Neurological Institute, Tianjin Institute of Immunology, State Key Laboratory of Experimental Hematology, International Joint Laboratory of Ocular Diseases, Ministry of Education, Haihe Laboratory of Cell Ecosystem, Tianjin Medical University General Hospital, Tianjin, 300052 China; 3 Department of Pharmacology, School of Basic Medical Science, Tianjin Medical University, Tianjin, China; 4 Department of Oncology, 363 Hospital, Chengdu, Sichuan China; 5 Department of Neurosurgery of First Affiliated Hospital, Fujian Medical University, Fuzhou, 350000 China; 6 China National Clinical Research Center for Neurological Diseases, Beijing Tiantan Hospital, Capital Medical University, Beijing, 100050 China

**Keywords:** Intracerebral hemorrhage, Hematoma, Perihematomal edema, CD8^+^ T cells, Neuroinflammation, Neuroimmunology, Mechanisms of disease, Translational immunology

## Abstract

**Abstract:**

Intracerebral hemorrhage (ICH) causes hematoma formation, leading to PHE, which is associated with leukocyte mobilization and increased inflammation at the site of brain injury. However, the fate of accumulated leukocytes within the hematoma and their impact on PHE expansion remain unknown. We performed single-cell immune profiling of hematoma cells from patients with acute ICH and reported a distinct phenotypic transformation of CD8^+^ T cells within the hematoma during the first 24 h after onset. In addition to enhanced IFN-γ production and migration capacity, these CD8^+^ T cells displayed remarkable glycolytic signatures. The metabolic fitness and functional reprogramming of hematomal CD8^+^ T cells are associated with the transcription factor FOXO1. Single-cell profiling of brain-infiltrating CD8^+^ T cells within the perihematomal tissues of ICH patients and cell culture assays revealed their capacity to activate microglia via the production of IFN-γ. Furthermore, the removal of hematomal CD8^+^ T cells reduced neuroinflammation, PHE expansion and neurological deficits in ICH mice. Thus, CD8^+^ T cells undergo metabolic and functional reprogramming within the hematoma during the acute phase of ICH, which contributes to PHE formation and neurological deterioration.

**Graphical Abstract:**

**Figure:**
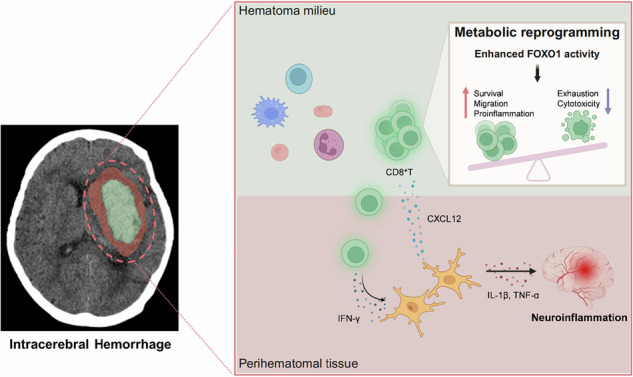
FOXO1-driven metabolic reprogramming of hematomal CD8^+^ T cells drives the expansion of perihematomal edema following intracerebral hemorrhage—mechanisms and clinical implications CD8^+^ T cells within the hematoma are transformed during the first 24 h after ICH onset. These hematomal CD8^+^ T cells can possess remarkable glycolytic signatures and enhanced IFN-γ production and migration capacity, which are associated with the transcription factor FOXO1. The infiltration of CD8^+^ T cells in the brain augments perihematomal edema and neurological deterioration. (Figure created with BioRender).

## Introduction

Intracerebral hemorrhage (ICH) accounts for up to 15% of all strokes and has high mortality and few treatment options [1, 2]. In addition to the primary mechanical destruction of neural structures caused by hematoma, ICH leads to the development of perihematomal edema (PHE), which induces secondary brain injury through disruption of the blood‒brain barrier (BBB) and destruction of adjacent tissue [3–5]. PHE occurs within 6 h following ICH onset, expands rapidly during the first 24 h and peaks at 3–7 days [6–8], leading to neurological deficits and long-term disability. However, the effectiveness of pharmacological interventions for ICH [8, 9] and removal of ICH hematoma via surgical evacuation or endoscopic clot aspiration with tissue plasminogen activator (tPA) remains to be fully evaluated in clinical studies [10]. Considering the contribution of PHE to clinical deterioration and its broad time window for intervention, PHE may represent an attractive therapeutic target in ICH.

ICH activates the immune system, which is characterized in part by glial activation and leukocyte infiltration into the perihematomal tissues within 24 h after onset [11–13]. The infiltrating leukocytes respond to damage signals and produce proinflammatory factors to initiate a neuroinflammatory cascade that contributes to the development of PHE and secondary brain injury during the acute stage of ICH [14]. The sources of brain-infiltrating leukocytes include immune cells that originate from the hematoma and those that actively migrate from the periphery across the BBB within 24 h after ICH onset [15, 16]. Preclinical and clinical studies have demonstrated that the inhibition of peripheral leukocytes that home to the ICH brain can reduce brain edema and improve neurological outcomes [14, 17]. However, the source and precise impact of infiltrating leukocytes on PHE development remain poorly understood.

Previous clinical studies suggest that efficient hematoma evacuation may alleviate PHE progression [18, 19]. Additionally, a subsequent study of the MISTIE III trial collected myeloid cells from hematoma clots aspirated with tPA for RNA sequencing and revealed altered myeloid cell activity within the hematoma over time in ICH patients [20]. Single-cell profiling of an ICH patient further provided a comprehensive view of immune cell dynamics, suggesting that leukocyte responses can be shaped in the hematoma milieu [21]. These findings suggest a potential role for leukocytes in hematoma during PHE progression. In addition to myeloid cells, lymphocytes such as CD8^+^ T cells are among the major components of the hematoma [22]. However, the fate of accumulated lymphocytes within the hematoma and their contribution to PHE remain unknown. In this context, several key questions remain unresolved. First, lymphocytes are critical contributors to neuroinflammation and ICH injury. Considering the swift infiltration of lymphocytes into the perihematomal tissue within 24 h after ICH, what are the immune features of lymphocytes within the hematoma prior to entering the perihematomal tissue? Second, previous characterization of myeloid cells within the hematoma utilized leukocytes collected from hematoma clot aspiration with exposure to tPA. As tPA is known to activate leukocytes, the features of lymphocytes within the hematoma without tPA exposure remain unclear. Third, what are the key cellular and molecular mechanisms that shape lymphocyte activity within the hematoma? Finally, to what extent and by what mechanisms do hematomal lymphocytes such as CD8^+^ T cells infiltrate the perihematomal tissue and contribute to PHE development? The present study seeks to address the above questions.

## Results

### Single-cell profiling reveals early accumulation of CD8^+^ T cells within the hematoma during the hyperacute phase of ICH in patients

**Fig. 1:**
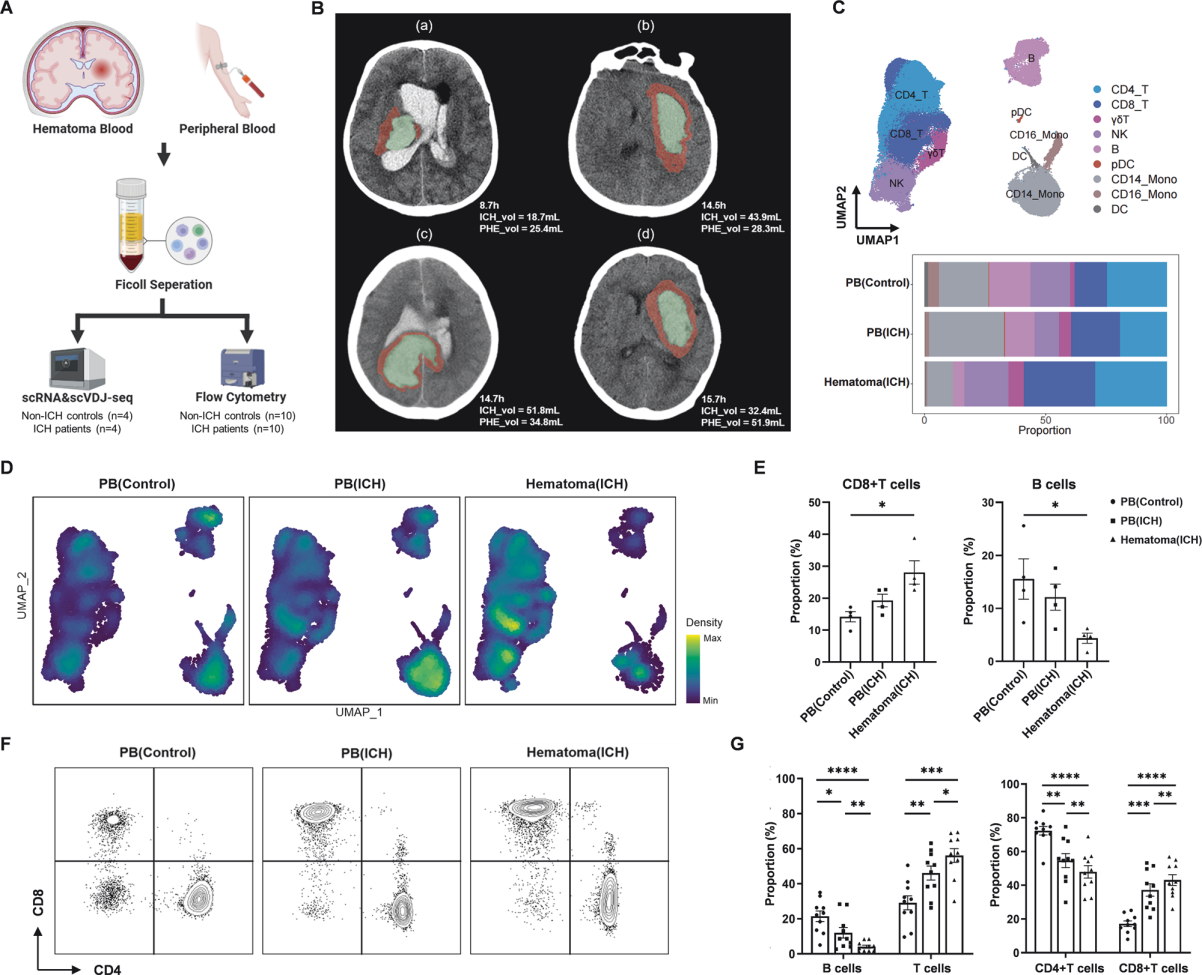
Accumulation of CD8^+^ T cells within the hematoma during the hyperacute phase of ICH. **A** Experimental design for single-cell sequencing and flow cytometry of immune cells from ICH patients and controls. Created with BioRender.com. **B** CT images of 4 ICH patients and quantification of PHE volume (in red) and ICH volume (in green). **C** UMAP plot of 79,721 cells derived from mononuclear cells of the peripheral blood and hematoma. Bar plots showing the proportions of different cell types in the indicated groups of subjects. **D** UMAP plot showing the cell density in the indicated groups of subjects. **E** Proportions of CD8^+^ T cells and B cells identified from the scRNA-seq data of the indicated groups of subjects. ICH patients: *n *= 4; controls: *n *= 4. **F** Flow cytometry plots showing the gating strategy of T cells for mononuclear cells in the peripheral blood and hematoma. **G** Bar graphs showing the proportions of B cells and T cells among total lymphocytes and the proportions of CD4^+^ T cells and CD8^+^ T cells among T cells in the indicated groups of subjects. ICH patients: *n *= 10; controls: *n *= 10. Mean ± SEM, *P < 0.05, **P < 0.01, ***P < 0.001, ****P < 0.0001. PB (Control): peripheral blood of controls; PB (ICH): peripheral blood of ICH patients; Hematoma (ICH): Hematoma blood of ICH patients

To characterize the features of immune cells within the hematoma during the hyperacute phase of ICH, we performed single-cell profiling of various immune cell subsets from peripheral blood and hematoma samples collected from ICH patients within the first 24 h after onset (Fig. 1A), in which CT scans revealed PHE formation (Fig. 1B). After quality control, a total of 79,721 immune cells were identified for subsequent analysis. Graph-based Louvain clustering algorithms were used to categorize all immune cells into 19 clusters and 9 cell types: CD4^+^ T cells (CD4_T), CD8^+^ T cells (CD8_T), γδ T cells (γδT), natural killer (NK), B cells (B), CD14^+^ monocytes (CD14_Mono), CD16^+^ monocytes (CD16_Mono), dendritic cells (DCs) and plasmacytoid dendritic cells (pDCs). Regions of the uniform manifold approximation and projection (UMAP) were occupied by cells of different immune cell types (Fig. 1C). The proportion of CD8^+^ T cells was markedly elevated within the hematoma (peripheral blood control vs. peripheral blood ICH vs. hematoma ICH: 14.2% vs. 19.3% vs. 28.1%) (Fig. 1C–E). Notably, the cell density plot revealed a prominent population of CD8^+^ T cells within the hematoma (Fig. 1D).

Next, we performed flow cytometry analysis to measure lymphocytes from patients and controls (Fig. 1F). Notably, we detected a marked decrease in B cells within the hematoma (peripheral blood control vs. peripheral blood ICH vs. hematoma ICH: 21.5% vs. 12.0% vs. 4.1%), accompanied by an increase in T cells (peripheral blood vs. peripheral blood ICH vs. hematoma ICH: 29.1% vs. 46.1% vs. 56.1%), which resulted from mainly CD8^+^ T cells (peripheral blood vs. hematoma ICH: 17.2% vs. 37.2% vs. 43.1%) (Fig. 1G). Together, these findings demonstrate that the composition of immune cells is altered within the hematoma during the hyperacute phase of ICH, which is characterized in part by a prominent increase in CD8^+^ T cells within the hematoma, suggesting a pivotal role of CD8^+^ T cells in shaping neuroinflammation and PHE development in ICH.

### The transcriptomic profiles of CD8^+^ T cells are altered within hematomas in patients with ICH

**Fig. 2:**
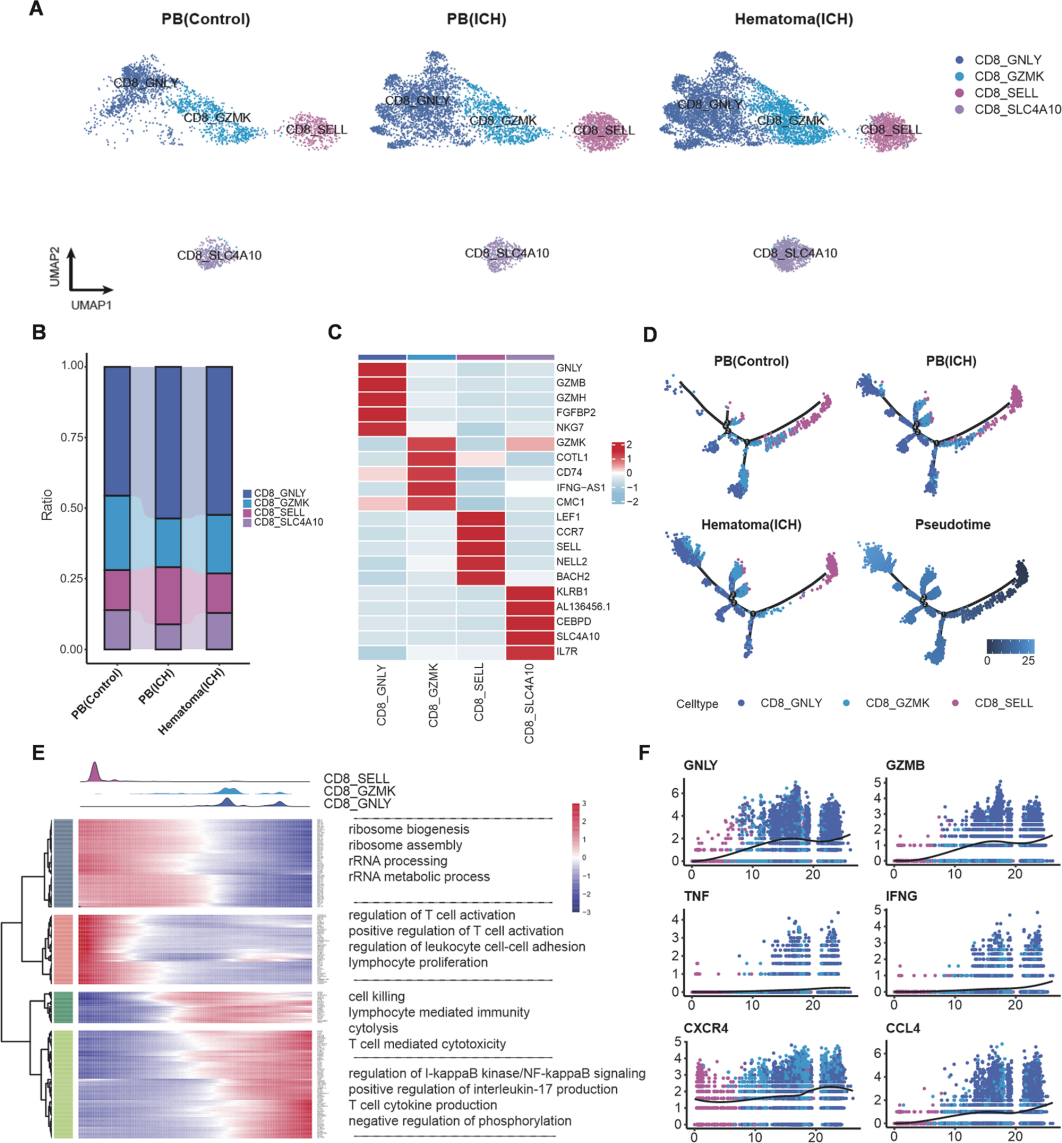
CD8^+^ T-cell subsets and their differentiation trajectories in the hematomas and peripheral blood of ICH patients. **A** UMAP of CD8^+^ T cells reclustered into 4 subpopulations: CD8_SELL, CD8_GZMK, CD8_GNLY and CD8_SLC4A10. **B** Bar plots showing the proportions of CD8^+^ T-cell subpopulations in the indicated groups of subjects. **C** Differential expression analysis comparing each cluster to others revealed distinct gene expression profiles, and the top genes from each cluster are displayed with gene names annotated on the right. **D** Trajectory of 4 clusters along pseudotime defined by Monocle2 in the indicated groups of subjects. **E** Heatmap showing differentially expressed genes during the trajectory and enriched GO terms for gene sets are represented on the right. **F** Averaged expression patterns of gene sets of interest along pseudotime

To characterize the transcriptomic profiles of hematomal CD8^+^ T cells in ICH, we performed transcriptomic profiling of CD8^+^ T-cell subsets (Fig. 2A–C), including CD8_SLC4A10 (which highly expresses mucosal-associated invariant T-cell markers such as *SLC4A10* and *KLRB1*), CD8_SELL (which highly express naïve T-cell markers such as *LEF1*, *SELL*, and *CCR7*), CD8_GZMK (which highly expresses effector-memory T-cell markers such as *GZMK* and *COTL1*), and CD8_GNLY (which highly expresses effector T-cell markers such as *GNLY*, *GZMB*, and *GZMH*) [23, 24]. We found that the CD8_GNLY subcluster was increased in the peripheral blood and hematoma of ICH patients (Fig. 2A, B), along with high expression of effector genes such as GNLY, GZMB and GZMH (Fig. 2C). To explore the dynamic transition of CD8^+^ T cells from the peripheral blood to the hematoma, we constructed a pseudotime map of the trajectory of the CD8^+^ T-cell state. We found that CD8^+^ T cells in ICH patients mainly differentiated toward the CD8_GNLY subpopulation, particularly in hematoma samples, whereas CD8^+^ T cells in controls were mainly retained in the naïve CD8_SELL state (Fig. 2D).

In the determination of gene expression patterns along with the trajectory of CD8^+^ T cells, we identified four distinct expression patterns (Fig. 2E). Functional enrichment analysis revealed genes related to diverse cellular functions (i.e., ribosome biogenesis, RNA processing and regulation of T-cell activation) that were highly expressed in the CD8_SELL state but were downregulated when those cells transitioned to the CD8_GNLY state. We also revealed the activation of several classical pathways, including the I-kappaB kinase/NF-kappaB signaling and T-cell cytokine production pathways, in the CD8_GNLY state (Fig. 2E). Moreover, we found that the expression of the GNLY, GZMB, IFNG and CXCR4 genes gradually increased during the transition from the CD8_SELL state to the CD8_GNLY state, suggesting increased effector function and chemotaxis activity (Fig. 2F).

Collectively, these results suggest that the transcriptomic features and effector functions of CD8^+^ T cells are dramatically altered within hematomas in patients with ICH.

### Metabolic reprogramming and related alterations in CD8^+^ T cells within the hematoma

**Fig. 3:**
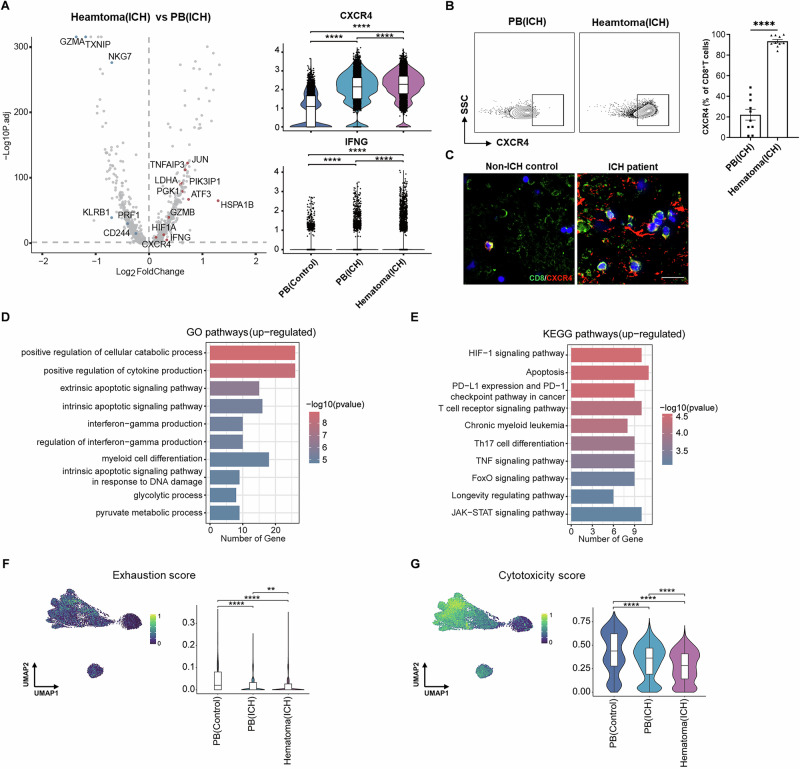
CD8^+^ T cells undergo metabolic and functional reprogramming within hematomas. **A** Volcano plots showing the differential expression of genes in the CD8_GNLY subpopulation between the hematoma and peripheral blood of ICH patients. Violin plots showing the expression of the indicated genes. **B** Flow cytometry plots showing the gating strategy of CXCR4 for CD8^+^ T cells in the peripheral blood and hematoma. Bar graphs showing the expression of CXCR4 in CD8^+^ T cells in the indicated groups of subjects. ICH patients: *n *= 10; controls: *n *= 10. **C** Immunostaining showing the expression of CXCR4 in brain-infiltrating CD8^+^ T cells in perihematomal brain tissue sections from patients with ICH. Scale bar: 20 µm. **D**, **E** Gene enrichment analyses of DEGs. **F**, **G** UMAP and violin plots showing the signature enrichment of CD8^+^ T-cell cytotoxicity and exhaustion in the indicated groups. Mean ± SEM, **P < 0.01, ****P < 0.0001

As previous analyses of immune cells within the hematoma were limited to >24 h after ICH onset with exposure to tPA [20], it remains unclear whether the activity of immune cells can be shaped within the hematoma as early as <24 h, i.e., during the hyperacute phase of ICH. To address this, we compared CD8^+^ T cells within the hematoma, mainly the CD8_GNLY subpopulation, with those in the periphery during the hyperacute phase of ICH. DEG analysis revealed the upregulation of multiple metabolic signatures (LDHA, PGK1, ATF3 and HIF1A) that are involved mainly in aerobic glycolysis, i.e., the Warburg effect (Fig. 3A). In addition, CD8^+^ T cells within the hematoma also exhibited partially upregulated inflammation signatures, such as IFNG and GZMB (Fig. 3A). As a hallmark of T-cell activation [25], a switch from oxidative phosphorylation (OXPHOS) to glycolysis along with the expression of proinflammatory cytokines such as interferon-γ (IFN-γ) suggests augmented T-cell responses within the hematoma. Our results suggest that CD8^+^ T cells undergo metabolic reprogramming within the hematoma and are rapidly activated during the hyperacute phase of ICH.

We also observed upregulation of the chemotaxis-related gene CXCR4 in hematomal CD8^+^ T cells, which was consistent with the results of flow cytometry (Fig. 3A, B). Additionally, immunostaining of perihematomal tissue sections from ICH patients revealed a marked increase in CD8^+^ T cells expressing CXCR4 (Fig. 3C). These findings suggest that CD8^+^ T cells within the hematoma possess enhanced migration capacity, which may allow them to infiltrate the surrounding brain tissue.

GO pathway enrichment analysis revealed that the CD8_GNLY subpopulation of CD8^+^ T cells in the hematoma presented an increase in genes related to the production of cytokines such as IFN-γ, along with the activation of glycolytic process pathways (Fig. 3D). KEGG pathway enrichment analysis demonstrated the activation of T-cell receptor signaling, apoptosis, HIF-1 signaling and TNF receptor signaling (Fig. 3E). Functional validation via a glycolytic stress test demonstrated that compared with peripheral CD8^+^ T cells, hematomal CD8^+^ T cells presented significantly higher extracellular acidification rates (ECARs) (Supplementary Fig. S1A). Furthermore, the inhibition of glycolysis with 2-deoxy-D-glucose (2-DG) markedly reduced IFN-γ secretion from hematomal CD8^+^ T cells (Supplementary Fig. S1B). These findings suggest that CD8^+^ T cells within the hematoma are held in a hypoxic state with increased glycolytic processes, implying that these cells exhibit a paradoxical functional state, where on the one hand, the apoptotic signal is increased, while on the other hand, they can maintain T-cell activation and produce proinflammatory cytokines.

We further compared the exhaustion and cytotoxicity scores of the CD8^+^ T cells. We found that the cytotoxicity score was lower and the exhaustion score was lower in the hematomal CD8^+^ T cells, suggesting that, under hypoxic conditions, T-cell cytotoxic effector functions are partially diminished to maintain T-cell survival (Fig. 3F, G).

Together, these findings suggest that hematomal CD8^+^ T cells are likely activated in metabolically stressful environments and undergo metabolic reprogramming to adopt distinct transcriptional profiles and functional features within the hematoma.

### FOXO1 controls the metabolic and functional state of CD8^+^ T cells within the hematoma

**Fig. 4:**
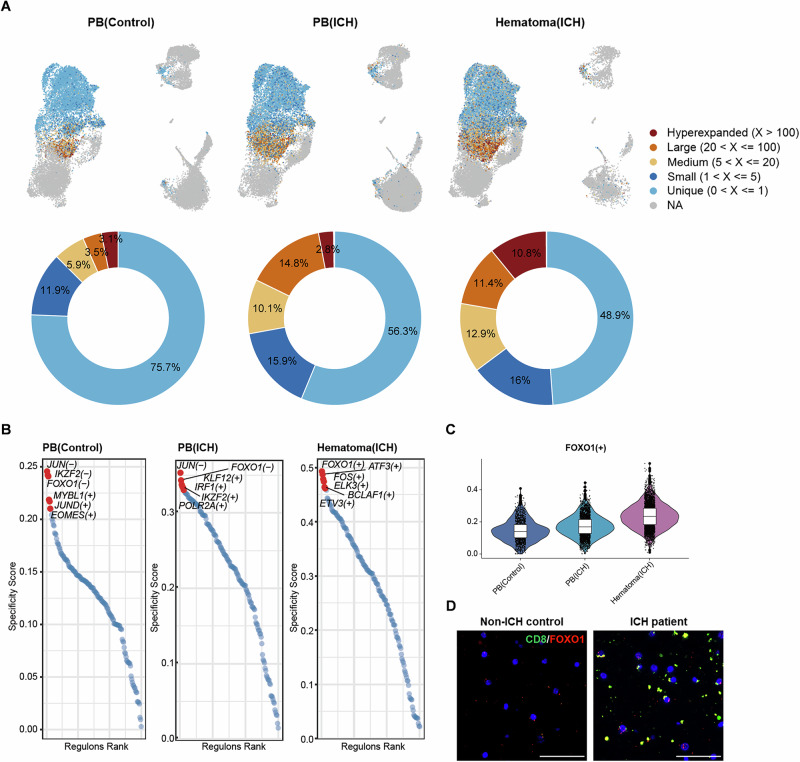
FOXO1 drives distinct phenotypic changes in CD8^+^ T cells. **A** TCR clone size colored in the UMAP plot and the proportion of clone size in the donut plot in the indicated groups of subjects. **B**, **C** Ranking plots and violin plots showing the regulon specificity score of FOXO1 expression in the indicated groups. **D** RNAscope analysis of FOXO1 expression in brain sections from patients with ICH and control subjects. Scale bar: 50 µm

Metabolic activity controls the activation of transcription factors (TFs), which guide T cells toward specific functional states and maintain their metabolic fitness [26, 27]. Evidence indicates that an immediate glycolytic switch contributes to the effector function of CD8^+^ T cells and their swift transition to an exhausted state [28]. In contrast, hematomal CD8^+^ T cells displayed partially diminished effector functions and a lower exhaustion state. We observed that CD8^+^ T cells within the hematoma displayed the greatest clonal size, suggesting that this unique CD8^+^ T-cell subset can adapt to the hematoma microenvironment (Fig. 4A).

To identify the key factors shaping the response of CD8^+^ T cells within the hematoma, we performed scVDJ-seq and found that T cells from the hematoma and peripheral blood exhibited significant homology (Supplementary Fig. S2). These findings suggest that the functional transformation of T cells is driven by the metabolic milieu within the hematoma rather than the inherent differences in the CD8^+^ T cells themselves. We subsequently measured the TFs in CD8^+^ T cells to identify the key TFs that determine the transformation of CD8^+^ T cells within the hematoma. We observed a significant increase in the expression of the TF FOXO1 in CD8^+^ T cells within the hematoma (Fig. 4B, C), which is closely associated with T-cell survival and stemness maintenance [29, 30].

Furthermore, we performed RNAscope using brain tissue sections from the perihematomal regions of ICH patients. We detected prominent infiltration of CD8^+^ cells that expressed FOXO1 within the perihematomal tissue (Fig. 4D). These findings suggest that the transformation of CD8^+^ T cells within the hematoma is associated with FOXO1. FOXO1-expressing CD8^+^ T cells can infiltrate perihematomal tissue during the early stages of ICH.

In addition, we performed additional analyses of myeloid subsets in the hematoma, focusing on their transcriptional profiles and potential interactions with CD8^+^ T cells (Supplementary Fig. S3). Cell–cell communication analysis revealed that among the myeloid subsets, CD14_Mono exhibited the most prominent interactions with CD8_T cells, which were mediated primarily through the SPP1 signaling pathway and chemokine–receptor pairs (Supplementary Fig. S3A, B). Further comparative analysis between hematomal and peripheral blood CD14_Mono data revealed that CD14_Mono upregulated SPP1 and chemokine-related genes (CXCL5, CXCL3 and CCL20), whereas the expression of myeloid-associated genes was downregulated (Supplementary Fig. S3C). Pathway enrichment analysis further revealed that T-cell differentiation, T-cell activation and T-cell receptor signaling were upregulated in the hematomal CD14_Mono population (Supplementary Fig. S3D, E). These results provide mechanistic insights into how myeloid subsets within the hematoma may interact with CD8⁺T cells to modulate local inflammation.

### CD8^+^ T cells activate microglia through the IFNG-IFNGR pathway in the perihematomal tissue

**Fig. 5:**
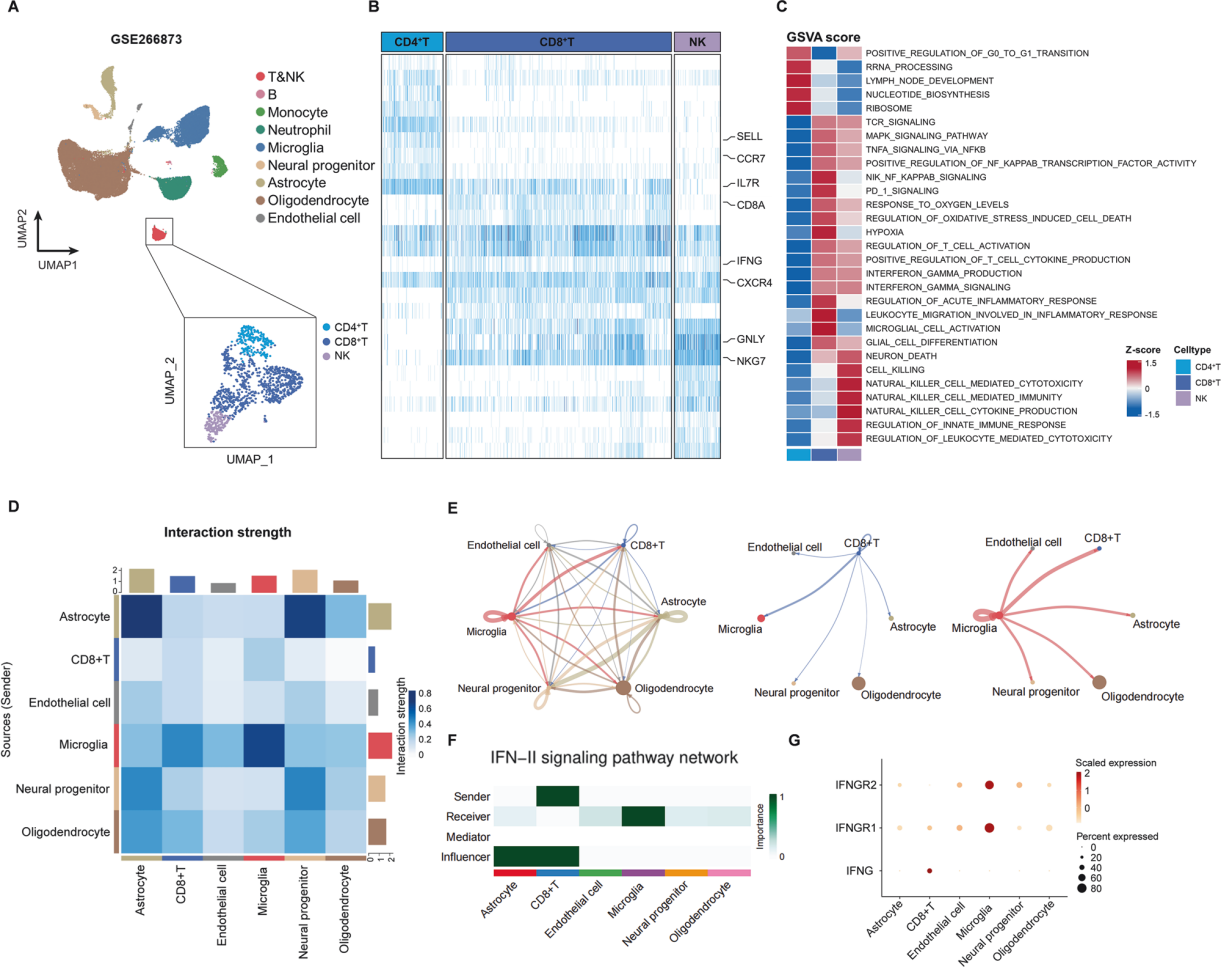
CD8^+^ T cells activate microglia in perihematomal tissues via the IFNG-IFNGR pathway. **A** UMAP plot of immune cells derived from PHE tissues of ICH patients on the basis of scRNA-seq data from the GEO database (GSE266873). **B** Heatmap displaying the expression levels of the top DEGs and key genes of interest across the T&NK subcluster. **C** GSVA analysis indicates enriched pathways of each subset of T&NK subclusters. **D**–**G** Intercellular interactions between CD8^+^ T cells and central immune cells in perihematomal tissues, revealing the IFNG-IFNGR signaling pathway

To determine the activity of the CD8^+^ T cells that infiltrate the perihematomal tissue, we performed single-cell analysis of the perihematomal tissues (GSE266873) [31]. We found that infiltrating lymphocytes within the perihematomal tissues were predominantly CD8^+^ T cells, which highly expressed IFNG, CXCR4 and GNLY (Fig. 5A, B). GSVA revealed that CD8^+^ T cells exhibited marked IFN-γ production and activity related to microglial activation (Fig. 5C). To determine the interactions between CD8^+^ T cells and brain-intrinsic cells, we performed a cellChat analysis and found that the interaction strength between CD8^+^ T cells and microglia was predominant (Fig. 5D, E). Similarly, microglia also have robust communication with CD8^+^ T cells, supporting a dramatic cellular interaction between CD8^+^ T cells and microglia. Through receptor‒ligand analysis, we further discovered that CD8^+^ T cells interact with microglia through the IFN-II signaling pathway as senders and receivers (Fig. 5F, G).

**Fig. 6:**
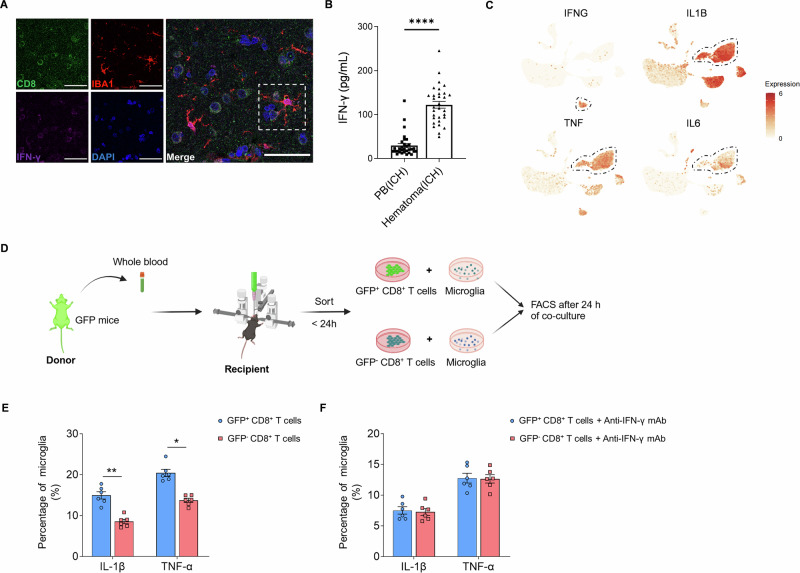
CD8^+^ T cells in the hematoma activate microglia in ICH mice. **A** Fluorescence colocalization analysis of CD8, IBA1, and IFN-γ in PHE regions from patients with ICH. Scale bar: 20 µm. **B** The bar graphs show the concentrations of IFN-γ in the peripheral blood and hematomas of ICH patients (*n *= 31). **C** Gene expression analysis of scRNA-seq datasets of PHE samples from ICH patients (GSE266873). **D** Schematic diagram of the in vitro coculture experiment. GFP reporter mice were used to track CD8+ T cells within the hematoma. **E**, **F** Flow cytometry analysis of IL-1β and TNF-α in cultured microglia collected from the indicated groups. *n *= 6 per group. Mean ± SEM, *P < 0.05, **P < 0.01, ****P < 0.0001. PB (ICH): peripheral blood of ICH patients; hematoma (ICH): hematoma blood of ICH patients

IFN-γ can prime microglia and promote their transformation toward a proinflammatory phenotype [32]. To examine this phenomenon in the context of ICH, we performed immunostaining of perihematomal tissues from ICH patients, which revealed that CD8^+^ T cells expressed IFN-γ and were located in close proximity to microglia (Fig. 6A). Consistently, we detected a marked increase in IFN-γ in the hematoma compared with that in the peripheral blood of ICH patients (Fig. 6B). To further delineate the cellular sources of inflammatory mediators, we analyzed the expression of *IFNG*, *IL-1B*, *TNF*, and *IL-6* in perihematomal tissue scRNA-seq data from ICH patients. The results showed that IFNG was expressed primarily by CD8^+^ T cells, whereas microglia predominantly expressed IL1B and TNF (Fig. 6C), which is consistent with prior studies [31].

To test whether CD8^+^ T cells can directly modulate microglial activity, we cocultured microglia and CD8^+^ T cells from the peripheral blood and hematoma (Fig. 6D). After 24 h of coculture, coculture with hematoma-derived CD8^+^ T cells led to a marked increase in the number of microglia that produced inflammatory factors compared with that in CD8^+^ T cells derived from peripheral blood (Fig. 6E). Moreover, neutralization of IFN-γ with an anti-IFN-γ mAb led to suppressed transformation of microglia toward the proinflammatory phenotype induced by coculture with hematoma-derived CD8^+^ T cells (Fig. 6F). These findings suggest that CD8^+^ T-cell-derived IFN-γ can augment the inflammatory activity of microglia.

### Removal of CD8^+^ T cells or genetic deletion of FOXO1 in hematomal CD8^+^ T cells reduces PHE following ICH

**Fig. 7:**
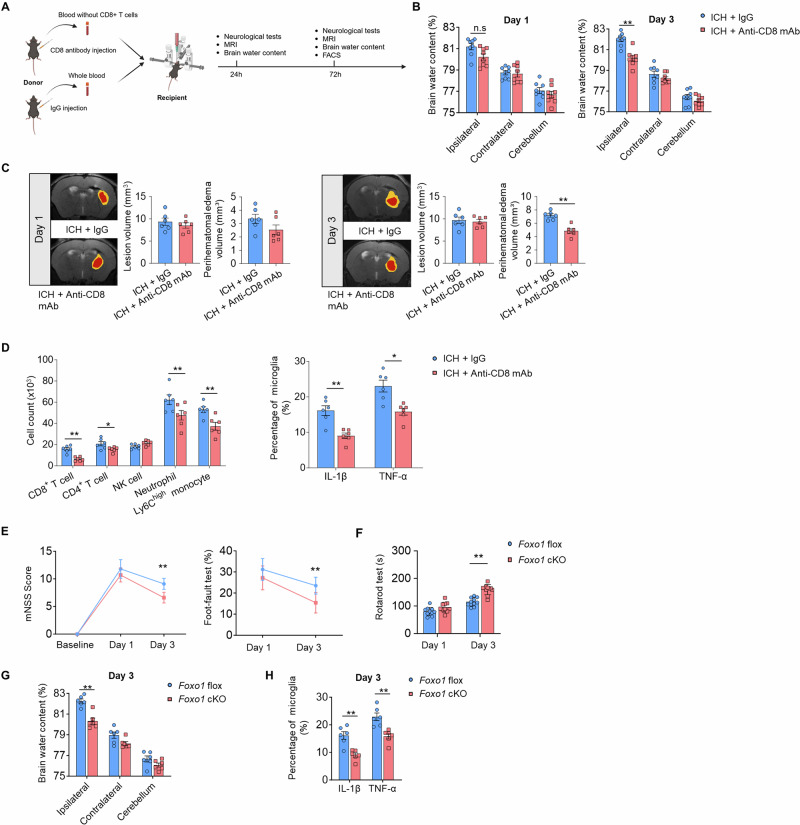
Removal of CD8^+^ T cells or genetic deletion of FOXO1 in hematomal CD8^+^ T cells attenuated neurological deficits and PHE in ICH mice. **A** Schematic showing the removal of hematomal CD8^+^ T cells and the experimental design for ICH mice. **B** Brain water content in the ipsilateral, contralateral and cerebellar regions on days 1 and 3 after ICH. *n *= 8 per group. **C** Quantitative analysis of lesion and PHE volume via MRI at days 1 and 3 after ICH. *n *= 6 per group. **D** Brain-infiltrating immune cell subsets and their expression of IL-1β and TNF-α in microglia from the indicated groups of ICH mice. *n *= 6 per group. **E**, **F** Neurological deficits were evaluated by the mNSS, foot fault test and rotarod test at the indicated time points after ICH. *n *= 10 per group. **G** Brian water content in the ipsilateral, contralateral and cerebellar regions on day 3 after ICH. *n *= 6 per group. **H** Microglia-derived proinflammatory factor levels in the indicated groups at 3 days after ICH. *n *= 6 per group. Mean ± SEM, *P < 0.05, **P < 0.01

To investigate the impact of hematomal CD8^+^ T cells on ICH injury, we intracerebrally injected donor peripheral blood without CD8^+^ T cells or with CD8^+^ T cells into the brain parenchyma of recipient mice (Fig. 7A). For CD8^+^ T-cell depletion, we injected an anti-mouse CD8a antibody intraperitoneally one day prior to ICH induction and analyzed the peripheral blood via flow cytometry before blood injection. The flow cytometry results revealed that the percentage of CD8^+^ T cells in the peripheral blood was significantly reduced, indicating successful depletion of CD8^+^ T cells (Supplementary Fig. S4A, B). Compared with ICH model mice, ICH model mice receiving intracerebral injections of peripheral blood without CD8^+^ T cells presented reduced PHE volume and neurological deficits (Fig. 7B, C, Supplementary Fig. S5A), underscoring the detrimental role of CD8^+^ T cells in exacerbating PHE expansion and neurological deterioration during the hyperacute phase of ICH.

To determine the impact of hematomal CD8^+^ T cells on neuroinflammation, we assessed brain-infiltrating leukocytes and microglial activation in ICH mice induced by intracerebral injection of peripheral blood without CD8^+^ T cells (Supplementary Fig. S5B), and we detected a marked decrease in infiltrating T cells and myeloid cells, as well as microglia expressing proinflammatory factors, compared with those in ICH mice receiving peripheral blood with CD8^+^ T cells (Fig. 7D). Using the CSF1R inhibitor PLX5622, we successfully depleted microglia within the ICH brain (Supplementary Fig. S4C, D) and reported a similar extent of ICH injury in groups of ICH mice induced by the injection of peripheral blood without CD8^+^ T cells and those with CD8^+^ T cells (Supplementary Fig. S5C, D). These findings suggest that hematomal CD8^+^ T cells contribute to PHE and ICH injury, a process involving microglia.

Additionally, we generated CD8⁺ T-cell–specific FOXO1 conditional knockout mice (*Cd8*^*Cre/+*^*; Foxo1*^*flox/flox*^, abbreviated as *Foxo1* cKO) and induced ICH. Our results revealed that the *Foxo1* cKO mice presented markedly alleviated neurological deficits, brain water content and microglia-derived proinflammatory factor levels (Fig. 7E–H). These findings suggest that Foxo1 in CD8^+^ T cells plays a crucial role in promoting microglial activation and subsequent neuroinflammation and brain edema following ICH.

## Discussion

This study provides the first definitive evidence that CD8^+^ T cells swiftly accumulate in the hematoma within the first 24 h after ICH and contribute to PHE during the hyperacute phase of ICH. CD8^+^ T cells, particularly FOXO1-driven CD8^+^ T cells, were markedly increased within the hematoma and displayed distinct features compared with those in the periphery. Within the hematoma, we identified a notable trend of CD8^+^ T cells transforming toward the CD8_GNLY subpopulation in patients with ICH, whereas the CD8^+^ T cells in controls were mainly retained in the naïve CD8_SELL state. Additionally, we observed extensive metabolic reprogramming of hematomal CD8^+^ T cells within the first 24 h after ICH, which was characterized by a hypoxic state along with increased glycolytic processes. Notably, these hematomal CD8^+^ T cells exhibited a unique functional state characterized by diminished cytotoxic effector function, which may facilitate their survival and increase the production of proinflammatory cytokines. Furthermore, we identified FOXO1 as a master switch that controls the transformation of CD8^+^ T cells within the hematoma. Additionally, we provide novel evidence that hematomal CD8^+^ T cells infiltrate perihaematomal tissues and exacerbate PHE, a process involving their action on microglia. Together, our findings revealed a previously unrecognized role of hematomal CD8^+^ T cells in PHE formation, highlighting that CD8^+^ T cells can swiftly undergo metabolic and functional reprogramming within the hematoma to exacerbate neurological deterioration following ICH.

This study focused on immune cell subsets such as CD8^+^ T cells within the hematoma within the first 24 h after ICH, an area that has not been adequately investigated in previous studies. In contrast to previous studies that focused on the role and function of immune cells in the periphery or brain parenchyma, only a few studies have investigated immune cells within the hematoma after ICH [20, 21]. A recent study investigated the dynamics and features of hematomal myeloid cells following ICH. However, owing to the use of tPA to harvest immune cells from hematoma effluent, the collected immune cells may be activated by preexisting tPA exposure, leading to altered features and functions. As such, our understanding of the dynamics and characteristics of immune cells within the hematoma remains limited [33]. In our study, hematomal immune cells were collected without the use of tPA within 16 h after ICH onset, i.e., the hyperacute phase of ICH. The comprehensive characterization of the cellular and molecular features of hematomal immune cells, such as CD8^+^ T-cell subsets, fills this knowledge gap by providing a new understanding of the local immune response within the hematoma and its impact on PHE expansion during acute ICH.

The finding that CD8^+^ T cells display distinct features within the hematoma versus the periphery suggests that the fate and function of CD8^+^ T cells are determined by the hematomal milieu, in which FOXO1^+^CD8^+^ T cells undergo metabolic reprogramming. These findings highlight that CD8^+^ T cells can transform into distinct functional states within the hematoma versus the periphery, a process involving metabolic reprogramming, which has not been adequately investigated in previous studies [27].

The identification of FOXO1-driven CD8^+^ T cells that undergo metabolic reprogramming within the hematoma has not only revealed the impact of the hematoma microenvironment on T cells but also revealed a novel functional state of these cells in acute ICH. As a critical transcription factor that sustains T-cell activity, FOXO1 has been reported to increase T-cell stemness, metabolic fitness and effector function. Consistent with the findings of previous studies, our findings highlight the role of FOXO1-driven CD8^+^ T cells in acute ICH, which may serve as a marker to assess the detrimental impact of hematomal CD8^+^ T cells on ICH injury. Multiple upstream cues, including metabolic alterations and inflammatory cytokine signaling, have been reported to induce FOXO1 activation [34–36]. We propose that the elevated expression of FOXO1 in CD8⁺ T cells following ICH is largely driven by the hematoma microenvironment. On the one hand, we observed significant enrichment of HIF-1 signaling, together with increased glycolysis, suggesting that the metabolic milieu within the hematoma induces adaptive responses in CD8⁺ T cells, with FOXO1 acting as a key regulator of this process. On the other hand, the abundance of inflammatory cytokines in the ICH milieu could also further augment FOXO1 expression through canonical signaling cascades. Taken together, these factors likely converge to maintain the elevated expression of FOXO1 in CD8^+^ T cells following ICH, but further studies are needed to delineate the precise mechanisms underlying this regulation.

Conventional treatment protocols for ICH utilize hematoma volume as a guiding factor for surgical intervention, often opting for conservative management in the case of a small hematoma. Our findings suggest that immune cells can be transformed within the hematoma and possess prominent detrimental features to infiltrate the perihematomal tissues and exacerbate PHE, leading to neurological deterioration after acute ICH. Given the prolonged process of hematoma clearance, the persistent migration of detrimental CD8^+^ T cells from hematomas toward perihematomal tissues may hinder the resolution of brain inflammation, which may lead to chronic inflammation and impaired neurorepair after ICH. As a result, our findings not only provide a new mechanistic understanding of the evolution of neuroinflammation after ICH but also support the need for early hematoma evacuation. In addition, these considerations imply that patients with ICH may also benefit from pharmacological interventions targeting T cells during the early stage of ICH. This underscores the need for reappraisal of treatment strategies on the basis of immune cell dynamics rather than merely hematoma size to improve neurological outcomes in ICH patients.

Owing to the challenges of sample collection, we cannot obtain longitudinal data on the changes in immune cells within hematomas in patients with ICH. In addition, there is a lack of cell tracking experiments to elucidate the mechanisms of immune cell migration within the hematoma toward the brain parenchyma. Our current data support that functional crosstalk between T cells and microglia is mediated by IFN-γ. However, whether antigen-specific recognition underlies this interaction remains to be elucidated. Further studies employing in vitro stimulation with brain-derived antigens at different time points after ICH are needed to conclusively address the role of antigen-specific T-cell–microglia interactions in this context. Collectively, these limitations highlight the need for future studies to comprehensively investigate the dynamic immune response within hematoma and perihematomal tissues over the course of ICH.

In summary, our findings underscore the previously unrecognized role of hematomal CD8^+^ T cells that can migrate into perihematomal tissues to shape the inflammatory response, leading to exacerbated PHE and neurological deterioration following ICH. These findings underscore the need for hematoma evacuation following ICH and highlight T-cell modulation as a potential therapeutic option that awaits future advanced investigations.

## Methods

### Study design

The purpose of this study was to define distinct changes in immune cells within the hematoma during the first 24 h after ICH. Patient studies were carried out in accordance with the Helsinki Declaration. The inclusion of human subjects and research protocols were approved by the Ethics Committee of the Affiliated Hospital of Fujian Medical University. Informed written consent was obtained from all the subjects. The patient inclusion and exclusion criteria were as follows: (a) were more than 18 years old, (b) had indications for hematoma evacuation due to spontaneous ICH, and (c) underwent hematoma evacuation within 24 h after ICH onset. The exclusion criteria were (a) preexisting neurologic diseases; (b) acute myocardial infarction, trauma, immune disease or hematological system disease prior to ICH; (c) immunosuppressant or immunomodulatory therapy within the last 30 days; (d) clinical signs of infection on admission; and (e) severe cardiac, hepatic, renal, or pulmonary dysfunction. Hematoma and brain tissues from perihematomal regions were collected from patients with ICH who underwent hematoma evacuation surgery. To ensure consistency in blood sources, peripheral blood was collected from arterial cannulas employed for invasive blood pressure monitoring. Arterial blood from patients without neurological disorders or acute cardiac diseases (those who underwent angiography) was used as a control. Control brain tissues were obtained from patients who underwent intracranial surgery for vascular malformations without prior hemorrhage or for epilepsy. Only tissues that required resection to gain surgical access were collected, which would otherwise have been discarded. All the samples were processed and stored according to our biobank protocols (Ethics approval: MRCTA, ECFAH of FMU [2022]427). After quality control, hematoma and peripheral blood samples collected from 4 patients, along with peripheral blood samples from age-matched controls, were processed immediately for single-cell RNA sequencing and V(D)J sequencing via the 10x Genomics platform (Supplementary Table S1). Blood samples for flow cytometry were collected from 10 patients with ICH and 10 controls. There was no significant difference in the age of patients with ICH and controls (ICH vs control: 65.1 ± 2.4 vs 62.1 ± 4.0 years, *P* = 0.617).

### Sample collection and cell preparation

Hematoma samples were filtered through a 70 µm cell strainer (Falcon) to remove brain debris. Each sample was centrifuged at 1500 × *g* for 10 min at room temperature, and the supernatant aliquots were subsequently stored in cryogenic vials at −80 °C until use. Peripheral blood plasma was processed and stored following the same procedure. Mononuclear cells from the hematoma and peripheral blood were isolated via density gradient centrifugation via Ficoll‒Paque PLUS (Cytiva) according to the manufacturer’s protocol.

### Animals

All animal experiments were performed in accordance with the ARRIVE (Animal Research: Reporting In Vivo Experiments) guidelines and approved by the Committee on the Ethics of Animal Experiments of Tianjin Neurological Institute, Tianjin Medical University General Hospital. We chose 2–3-month-old adult mice for subsequent experiments. B6-GFP mice (stock No: 004353) were purchased from The Jackson Laboratory, and C57BL/6 mice were purchased from the Vital River Corporation. The Rosa26-Cd8a-Cre mouse strain (stock no: I001090) and Foxo1-flox mouse strain (stock no: S-CKO-12115) were purchased from Cyagen. The offspring from crossing these two mouse strains were used to generate a conditional knockout mouse model (*Cd8*^*Cre/+*^*; Foxo1*^*flox/flox*^, abbreviated as *Foxo1* cKO). All the mice were housed in a pathogen-free facility with a standardized light‒dark cycle and unlimited access to food and water.

### ICH model

ICH was induced via intracerebral injection of donor whole blood as previously described [37, 38]. Briefly, after anesthesia, recipient C57BL/6 mice were positioned in a stereotaxic apparatus. After exposure of the cranium, a burr hole was drilled at the right side of the skull (2.3 mm right and 0.5 mm forward to the bregma). Nonheparinized blood was withdrawn from the angular vein of donor mice. Using a two-step injection method, 5 μL of blood was first infused (3 mm depth below the surface of the skull), and then 25 μL of blood was infused (3.5 mm depth below the surface of the skull). The needle remained in place for 15 minutes post-injection to avoid overflow and then was slowly retrieved.

To investigate the role of hematomal CD8^+^ T cells in ICH, donor C57BL/6 mice were pretreated with intraperitoneal injections of an anti-mouse CD8a antibody (BE0061, BioXcell) for CD8^+^ T-cell depletion or a rat IgG2b isotype (BE0090, BioXcell) as a control [39]. For in vitro studies, B6-GFP mice were used as donors to enable tracing of hematomal CD8^+^ T cells.

To study the potential role of microglia following ICH, PLX5622 (Selleckchem, Houston, TX) was formulated in AIN-76A standard chow at a dose of 1.2 g of PLX5622 per kilogram of diet. Prior to ICH induction, six-week-old mice were fed either control chow or chow with PLX5622 for 14 consecutive days.

### Brain water content measurement

The brain water content was evaluated as previously reported [40]. In brief, the mice were euthanized, and the brains were dissected without perfusion. The wet weights of the contralateral hemisphere, ipsilateral hemisphere, and cerebellum (used as internal controls) were measured separately. The brain tissues were then dried at 100 °C for 24 h, after which their dry weights were measured again. The brain water content was calculated as the percentage of weight lost from wet to dry via the following formula: [(wet weight − dry weight)/wet weight] × 100%.

### Neurological assessment

Neurological function was assessed independently by two investigators who were blinded to the experimental groups on days 1 and 3 after ICH. The modified neurological severity score (mNSS), foot-fault test, and rotarod test were performed [41]. The neurological deficits of the mice were evaluated via the modified neurological scale (mNSS), which comprehensively evaluates motor, sensory, reflex, and balance functions via a battery of tests. The foot-fault test was used to assess motor function and limb coordination. The mice were placed on an elevated grid with square openings and allowed to move across the grid. The total number of steps it takes to cross the grid and the foot fault for each limb are quantified. A rotarod test was conducted to assess equilibrium behavior and locomotor function. The mice were subjected to the rotarod test after being trained for 3 days prior to ICH induction and then positioned in the center position on the accelerating rotating rod (accelerated from 5 rpm to 40 rpm for 90 s and then 40 rpm for up to 5 min). The latency to fall on the platform was recorded.

### MRI measurement of PHE

The brain lesions and PHE volume of the ICH mice were quantified with a 9.4-T MRI scanner as we previously described [42]. To determine the lesion and PHE, the scanners used susceptibility-weighted image sequences and T2-weighted image sequences, respectively. Using Medical Image Processing, Analysis, and Visualization software (MIPAV; NIH), the lesion and perihematomal edema volumes were manually marked on each slice, and the areas in all slices were summed and multiplied by the section thickness.

### Cell culture

Microglia and CD8^+^ T cells were sorted from the recipients’ brains within 24 h after ICH via flow cytometry with a purity exceeding 95%. Microglia (CD45^int^CD11b^+^) were seeded in 24-well plates at a density of 5 × 10^4^ cells/well in DMEM/F12 supplemented with 10% FBS and 1% penicillin‒streptomycin. For coculture, hematomal CD8^+^ T cells (GFP^+^CD8^+^) and peripherally infiltrated CD8^+^ T cells (GFP^-^CD8^+^) were added to microglia-containing wells at a ratio of 1:2 (microglia:CD8^+^ T cells). After 24 h of coculture, the cells were processed for flow cytometry analysis.

### Library preparation and scRNA sequencing

In accordance with the manufacturer’s instructions, scRNA-seq and scVDJ-seq libraries were constructed via the single-cell 5’ library and Gel Bead Kit V2. In brief, single cells were resuspended at a concentration of 700 ~ 1200 cells/µL (viability ≥90%) and then loaded onto the 10× Chromium platform, followed by 5’ gene expression library and immune repertoire library preparation. The libraries were sequenced on an Illumina NovaSeq 6000 instrument using 150 paired-end reads with a sequencing depth of at least 50,000 reads per cell.

### Single-cell RNA sequencing

The reads of each gene in the scRNA-seq and scVDJ-seq data were quantified against the GRCh38 human reference genome via the Cell Ranger (version 7.1) single-cell software suite. Seurat objects were generated via Seurat 4.4.0, and the quality control criteria were that the number of genes detected per cell was less than 4000, the proportion of mitochondrial genes was less than 15%, and the proportion of ribosome genes was less than 40%. Next, NormalizeData were applied for normalization; FindVariableFeatures was used to find highly variable genes; ScaleData were converted to Z scores for PCA dimensionality reduction; RunHarmony was used to remove batch effects; the FindNeighbors and FindClusters functions were used for cell cluster analysis; and RunUMAP was used for nonlinear dimensionality reduction and visualization.

To integrate data sets from different batches into a shared space for unsupervised clustering, we used the harmony algorithm for batch effect correction. The highly expressed genes in each cluster were identified according to FindAllMarkers, and the top genes were regarded as marker genes. We annotated the cell types for all the clusters on the basis of known marker genes. Differentially expressed genes (DEGs) between the patient and control groups were identified via the FindMarkers function. Gene set scores were calculated for individual cells via the AddModuleScore function, which computes the average expression level of specified gene sets and normalizes them via aggregated control feature expression. The cytotoxicity score was defined via cytotoxicity-associated genes (*PRF1, IFNG, GNLY, NKG7, GZMA, GZMB, GZMH, GZMK, GZMM, KLRK1, KLRB1, KLRD1, FCGR3A, FGFBP2, ZEB2, CTSW, and CST7*), and the exhaustion score was defined via exhaustion-associated genes (*PDCD1, CTLA4, LAG3, CD160, TIGIT, HAVCR2, and CD244*) [43, 44]. Developmental trajectories were analyzed with Monocle (version 2.26.0). Gene Ontology (GO) enrichment and pathway enrichment analyses were conducted via clusterProfiler (version 4.8.3). Single cells were ordered in pseudotime to map cell differentiation. Single-cell regulatory network inference and clustering (SCENIC) was employed to identify gene regulatory networks and assess their activity within different clusters. TCR clonotype tracking and abundance calculations were performed via scRepertoire (version 2.0.4). Gene set variation analysis (GSVA) was performed for pathway enrichment using the gene sets downloaded from MsigDB (version 7.5.1). Cell‒cell communication was inferred via CellChat (version 1.6.1), and the interaction intensity of the information flow between cells was calculated.

### Flow cytometry

Mononuclear hematoma and peripheral blood cells were obtained via density gradient centrifugation, after which single cells were suspended in 1% bovine serum albumin. The following fluorescently conjugated antibodies were used for expression analysis of the human lymphocytes by flow cytometry: CD3-BV510 (564997, BD Biosciences), CD19-Percp (563109, BD Biosciences), CD4-FITC (344604, Biolegend), CD8a-BV650 (563821, BD Biosciences), and CXCR4-APC (555976, BD Biosciences). For cell proportion analysis of the mice, the following antibodies were used: CD3-APC (100236, BioLegend), CD4-BV711 (100549, BioLegend), CD8a-BV421 (100753, BioLegend), NK1.1-PE/Cy5 (108716, BioLegend), CD11b-PE/Cy7 (101216, BioLegend), Ly6G-BV510 (127633, BioLegend), Ly6C-BV785 (128041, BioLegend), CD45-PerCP-Cy5.5 (103132, BioLegend), IL-1β-PE (12-7114-82, eBioscience), and TNF-α-BV605 (506329, BioLegend). Cytometric staining was performed according to the manufacturer’s protocol. In brief, the cells were stained with surface antibodies for 30 minutes on ice in the dark. Single-stain reference controls were created via a negative control compensation particles set (BD Biosciences). Flow cytometry data were acquired by Celesta (BD Biosciences) and analyzed via FlowJo software (v10.8.1).

### Immunostaining

Immunostaining of the sections was performed as previously described [45]. Briefly, immunohistochemistry was performed on 6-µm frozen sections. After permeabilization and blocking, the sections were incubated with primary antibodies overnight at 4 °C. Following three rinses with PBS, the sections were incubated with species-appropriate fluorochrome-conjugated secondary antibodies for 60 min at room temperature. After another three rinses with PBS, the sections were subsequently mounted with an anti-fluorescence quenching agent containing 4′,6-diamidino-2-phenylindole (DAPI) for nuclear staining. The following primary antibodies were used: goat anti-Iba1 (ab5076, Abcam), mouse anti-CD8a (66868-1-Ig, Proteintech), rabbit anti-CXCR4 (ab124824, Abcam), and rat anti-IFNγ (14-7311-81, Invitrogen).

### RNA scope

In situ hybridization (ISH) assays were performed with RNAscope technology utilizing the RNAscope Fluorescent Multiplex Kit v.2 and an RNA‒Protein codetection ancillary kit (323100 and 323180, Advanced Cell Diagnostics ACD) in reference to the manufacturer’s instructions: Multiplex Fluorescent Reagent Kit v2 User Manual (323100-USM, ACD) and RNAscope® Multiplex Fluorescent v2 Assay combined with Immunofluorescence-Integrated Co-Detection Workflow.

Briefly, paraffin sections of brain tissue were heated at 60 °C for 30 min, followed by deparaffinization in xylene and washing with 100% ethanol. The sections were incubated in hydrogen peroxide for 10 min at room temperature. The tissue was pretreated by steaming in Co-Detection Target Retrieval solution for 15 min, followed by incubation with the mouse anti-CD8a antibody clone 1G2B10 (66868-1-Ig, Proteintech) at 4 °C overnight. After primary antibody incubation, the sections were fixed in 10% neutral buffered formalin solution for 30 min at RT and treated with Protease Plus for 30 min at 40 °C. RNAscope Probe-Hs-FOXO1 (470621, ACD) was hybridized at 40 °C for 2 h. After probe labeling, the sections were subjected to amplification steps (AMP1--3) and conjugated to fluorescent TSA Vivid 570 (PG-323272, Tocris). At the end of the RNAscope procedures, the sections were treated with donkey anti-mouse IgG (H + L) conjugated to Alexa Fluor 488 (715-545-150, Jackson ImmunoResearch) for 30 min at room temperature. Cover slips were applied after the nuclei were counterstained with the DAPI solution provided in the RNAscope multiplex kit. Confocal images were taken on a Zeiss LSM 880.

### ECAR measurements

A Seahorse XF Glycolysis Stress Test Kit (#103020-100, Agilent) was used to determine the extracellular acidification rate (ECAR) via a Seahorse XFe24 analyzer. CD8^+^ T cells from ICH patients were isolated via a CD8^+^ T-Cell Isolation Kit (#130--096--495, Miltenyi) and cultured in X-VIVO 15 serum-free medium (#02--053Q, Lonza) for recovery prior to analysis. Following the protocols, the cells were then resuspended in “Seahorse Medium” and seeded into XF24 cell culture microplates at a density of 1 × 10^5^ cells/well. The ECAR was evaluated by sequentially treating the cells with 10 mM glucose, 1 μM oligomycin, and 50 mM 2-DG. The impact of each addition on the ECAR was measured, and the data were again normalized to the cell count. Basal glycolysis was determined by the difference in the readings before and after glucose addition, and the glycolytic capacity was determined as the difference in the ECAR between glucose addition and oligomycin treatment.

### Cytokine assay

Briefly, supernatant aliquots from the hematoma and matched peripheral blood were collected for cytokine detection. Cytokine levels were measured via a Luminex 200 system (Luminex) according to the manufacturer’s instructions and conducted by LabEx (Shanghai).

### Enzyme-linked immunosorbent assay

For the glycolysis inhibition experiment, CD8^+^ T cells from ICH patients were isolated and then cultured in X-VIVO 15 serum-free medium at 37 °C and 5% CO_2_ for 24 h, with or without the addition of 2-deoxy-D-glucose (2-DG). The culture supernatants were collected after centrifugation to remove the cellular debris. The levels of IFN-γ were measured via ELISA kits (Elabscience, China). The OD value was measured at 450 nm, and the concentrations were calculated on the basis of the standard curve.

### Statistical analysis

GraphPad Prism version 8.3.0 and R version 4.3.0 were used for the statistical analyses. Power analysis and sample size calculations were performed on the basis of previous experience with the respective tests, variability of the assays and interindividual differences among experimental groups. All the experiments presented in this study were repeated at least three times. Two-tailed unpaired Student’s *t* tests were used to compare data from two independent groups. For comparisons of two or more variables among multiple groups, two-way ANOVA followed by Tukey’s post hoc test was used. *P* < 0.05 was considered to indicate statistical significance. The data are expressed as the mean ± SEM.

## Supplementary information

Supplementary Figures
