# Supplementary material for: FOXO1-driven metabolic reprogramming of hematomal CD8+ T cells drives the expansion of perihematomal edema following intracerebral hemorrhage
Source: Cell Mol Immunol. 2025 Nov 14;22(12):1629–41. doi: 10.1038/s41423-025-01363-x (PMC12660799; doi:10.1038/s41423-025-01363-x)
Supplement: Supplementary file 1 — Supplementary Figures [file 41423_2025_1363_MOESM1_ESM.docx]

**Supplementary Figures**

**
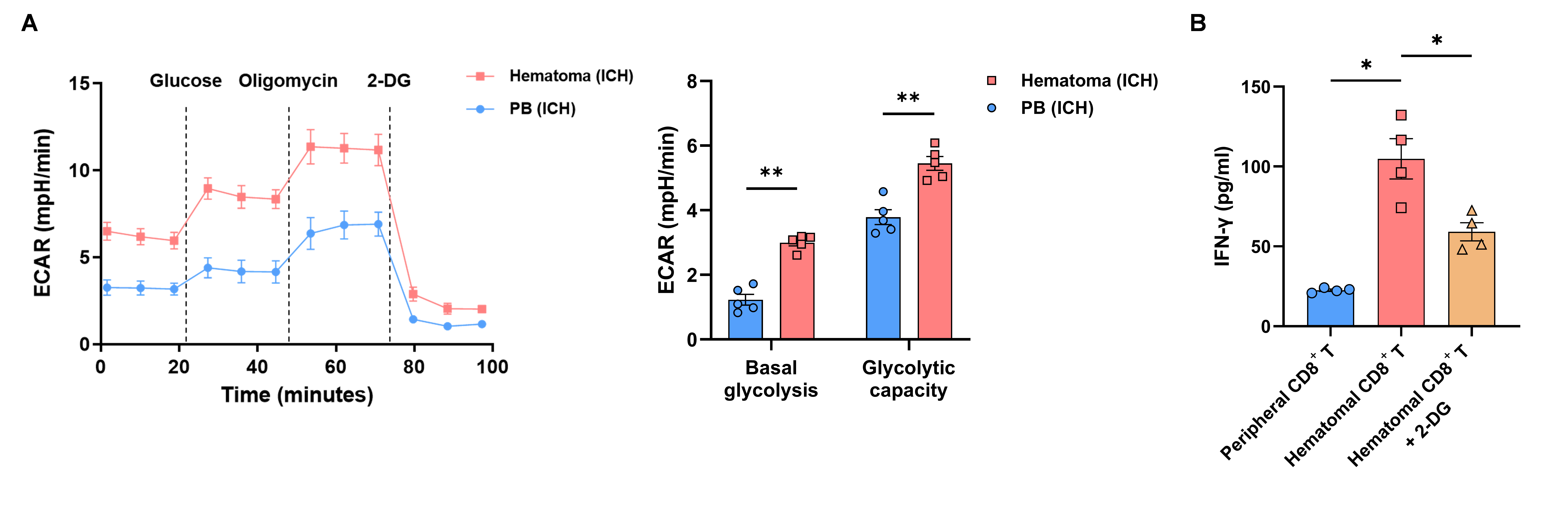
**

**Figure S1. Glycolytic activity and functional impact of CD8^+^T cells in ICH.**

**(A)** Seahorse glycolytic stress test of CD8^+^ T cells from hematoma and peripheral blood (n = 5). **(B)** IFN-γ production by CD8^+^ T cells from peripheral blood, hematoma, and treated with the glycolysis inhibitor 2-DG (2.5 mM) (n = 4). Data are presented as mean ± SEM. *p < 0.05, **p < 0.01.

**
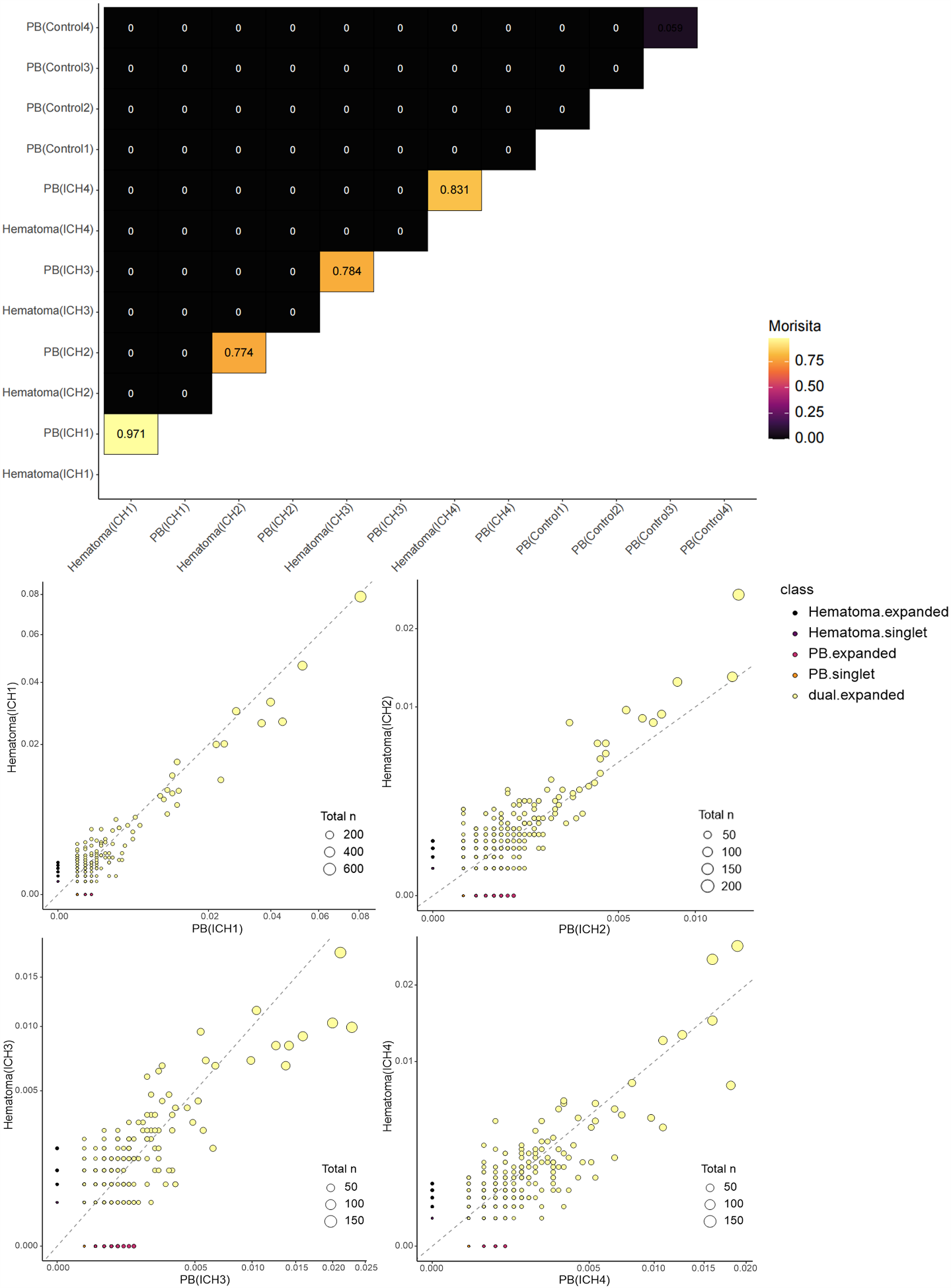
**

**Figure S2.** **T cells from the hematoma and peripheral blood exhibit significant homology.**

**F****igure S3. Interactions between myeloid subsets and CD8^+^ T cells within the hematoma, and their transcriptional profiles.**


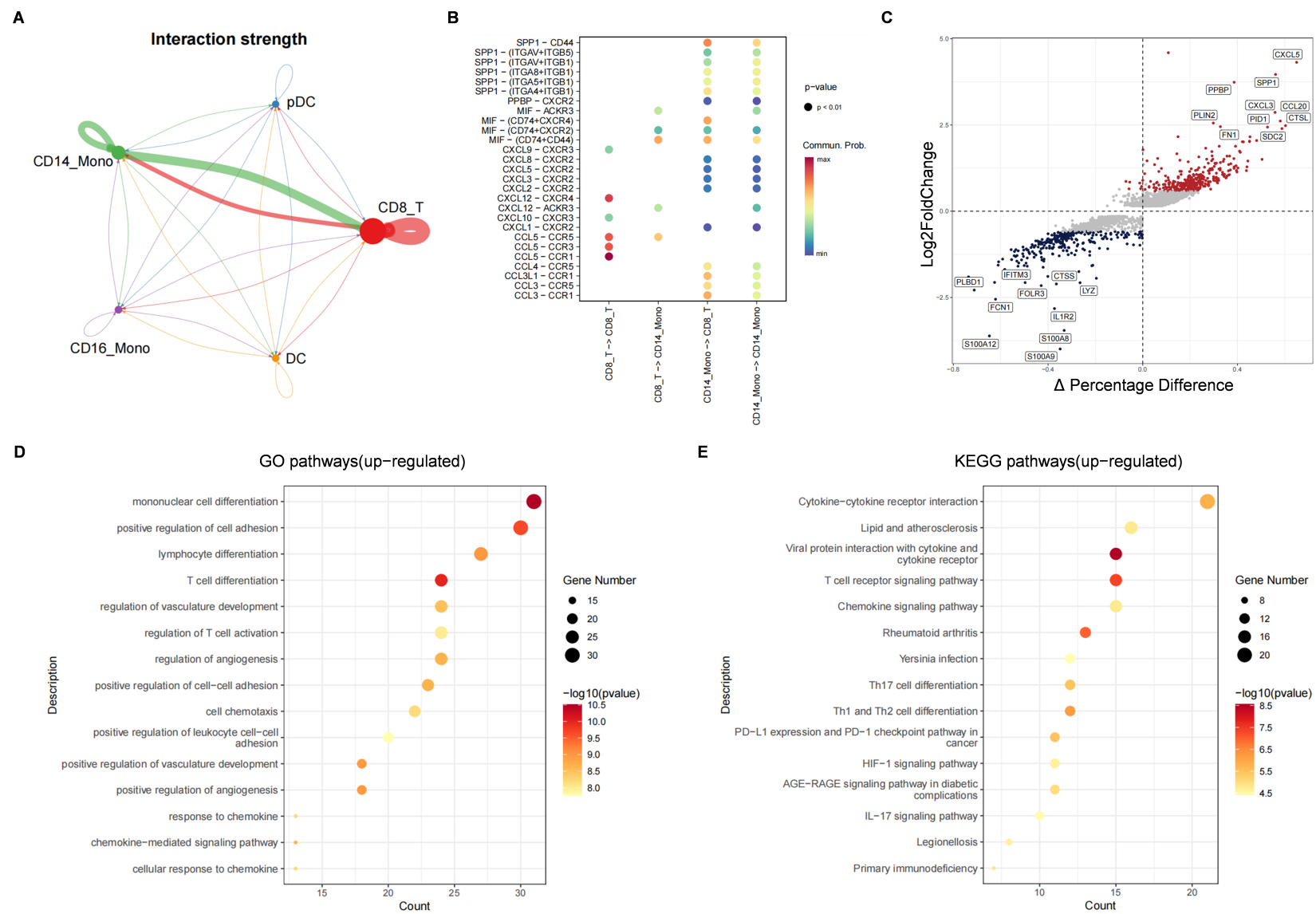


(**A-B**) Intercellular interactions between myeloid subsets and CD8^+^ T cells within the hematoma. (**C**) Differential gene expression (DEG) analysis of CD14_Mono subset using the Log2FoldChange expression versus the difference in the percentage of cells expressing the gene comparing hematoma versus peripheral blood cells (Δ Percentage Difference). (**D-E**) Gene enrichment analyses of up-regulated genes in hematoma.


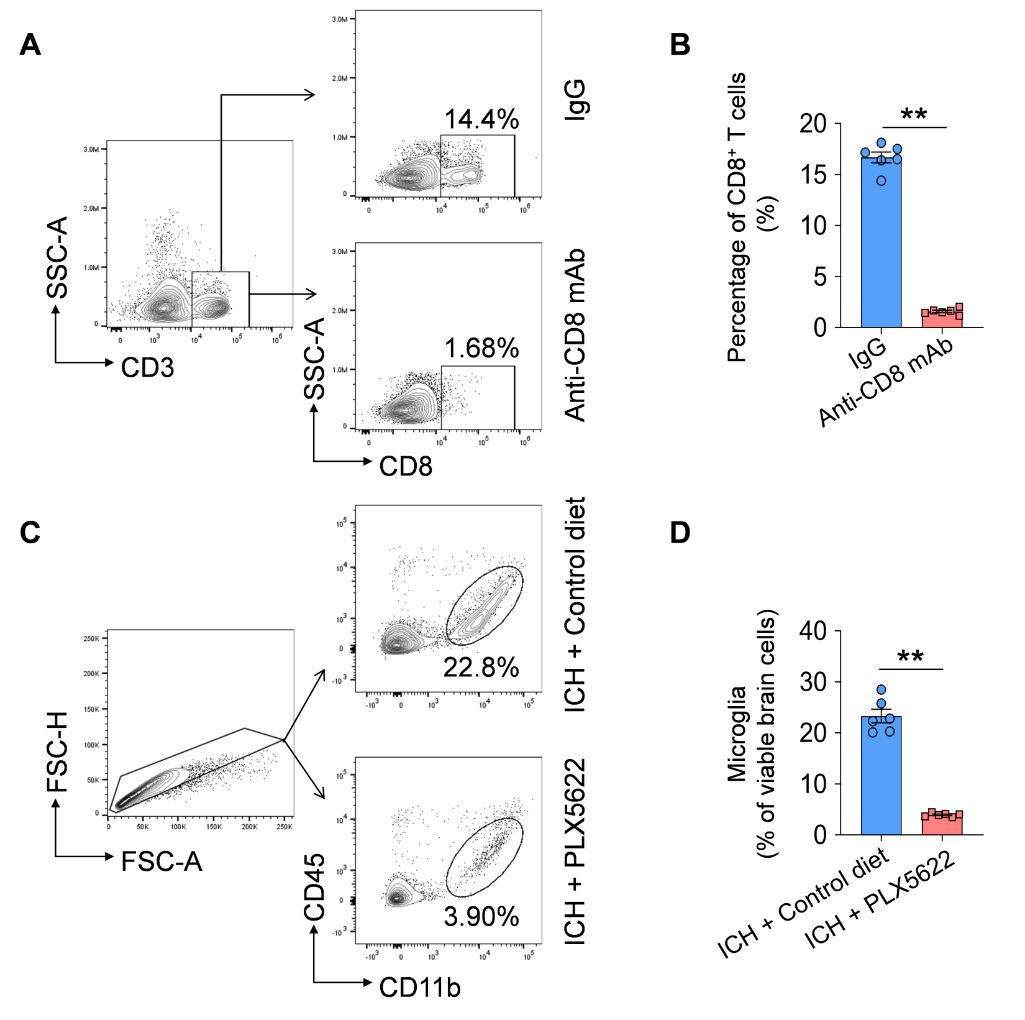
**Figure S4. CD8^+^ T cell and microglia were effectively eliminated by antibody or CSF1R inhibitor.**

**(A)** Flow cytometry plots showing the gating strategy of CD8^+^ T cells of peripheral blood. (**B**) Bar graph showing proportions of CD8^+^ T cells in T cells in indicated groups of subjects. (**C**) Flow cytometry plots showing the gating strategy of microglia of the brain. (**D**) Bar graph showing proportions of CD45^int^ CD11b^+^ microglia in mice receiving PLX5622 or control diet. n = 6 per group. Data are presented as mean ± SEM. **p < 0.01

**Figure S5. Removal of hematomal CD8^+^ T cells attenuates neurological deficits and brain inflammation following ICH in mice.**


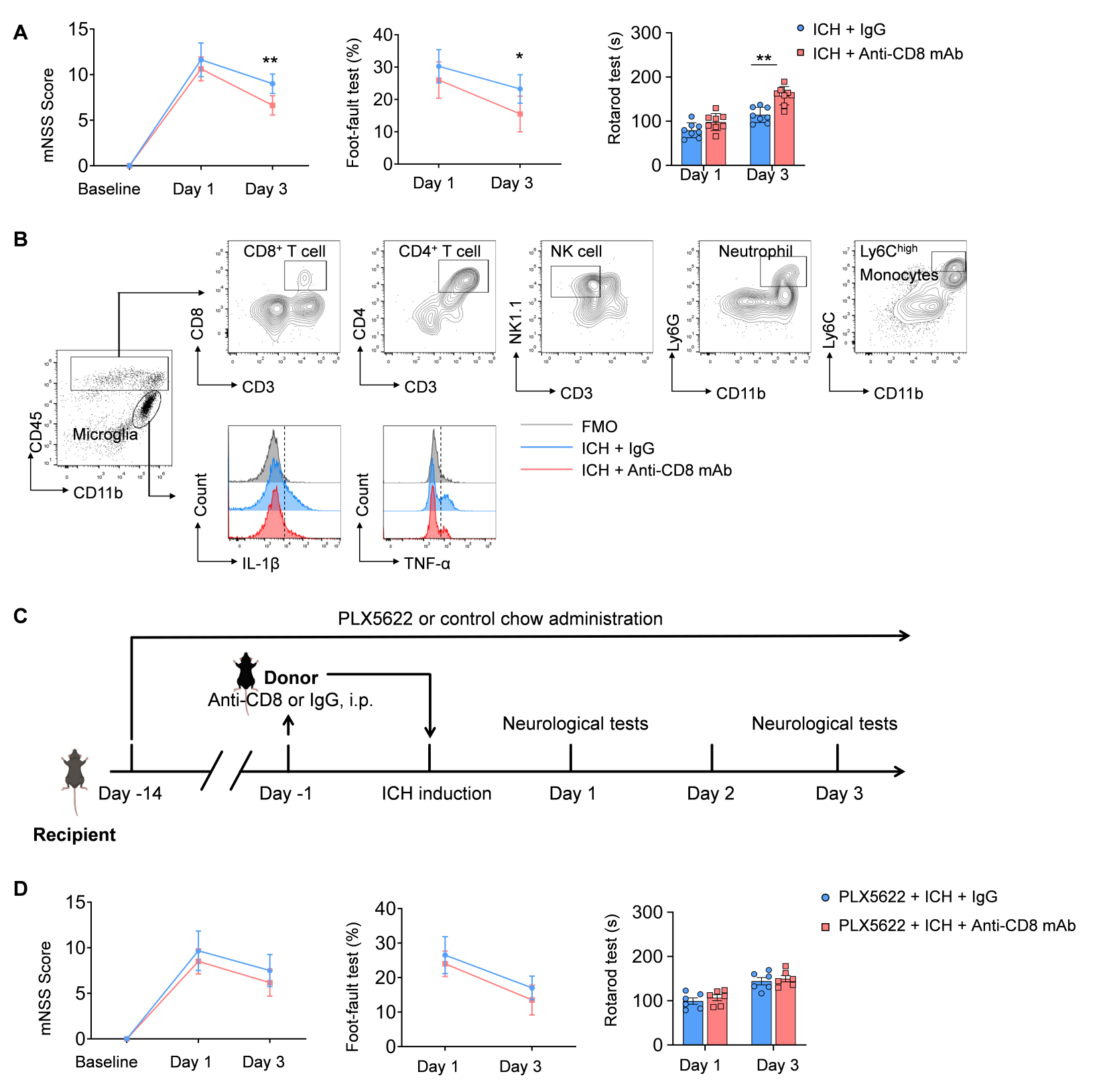


(**A**) Summarized results of neurological deficits at indicated time points after ICH. n = 8 mice per group. (**B**) Flow cytometry plots showing the gating strategy of brain-infiltrating leukocytes and microglia in ICH mice. (**C**) Schematic showing the experiment design to deplete microglia in ICH mice using a CSF1R inhibitor PLX5622. (**D**) Neurological deficits evaluated by mNSS, foot-fault test and rotarod test at indicated time points from indicated groups. n = 6 per group. Mean ± SEM, * P < 0.05, ** P < 0.01

**Table S1. Patient characteristics**

| Patient ID | Age  (y) | sex | Hypertension history | Craniotomy time after onset  (h) | Sampling time after onset  (h) | GCS score | ICH location | ICH volume  (mL) | PHE volume  (mL) |
| --- | --- | --- | --- | --- | --- | --- | --- | --- | --- |
| ICH 1 | 39 | Male | No | 5.2 | 8.7 | 2T | basal ganglia | 18.7 | 25.4 |
| ICH 2 | 29 | Male | Yes | 11.3 | 14.5 | 4T | basal ganglia | 43.9 | 28.3 |
| ICH 3 | 61 | Male | Yes | 11.8 | 14.7 | 3T | frontal parietal | 51.8 | 34.8 |
| ICH 4 | 58 | Female | Yes | 14.9 | 15.7 | 12 | basal ganglia | 32.4 | 51.9 |
| Control 1 | 33 | Male | Yes | - | - | - | - | - | - |
| Control 2 | 56 | Female | Yes | - | - | - | - | - | - |
| Control 3 | 31 | Male | No | - | - | - | - | - | - |
| Control 4 | 59 | Female | Yes | - | - | - | - | - | - |
